# Cryo-EM structure of a phosphotransferase system glucose transporter stalled in an intermediate conformation

**DOI:** 10.1016/j.yjsbx.2025.100124

**Published:** 2025-03-05

**Authors:** Patrick Roth, Dimitrios Fotiadis

**Affiliations:** Institute of Biochemistry and Molecular Medicine, Medical Faculty, University of Bern, Bern, Switzerland

**Keywords:** Cryo-electron microscopy, Elevator-type mechanism, Glucose transporter, Intermediate conformation, Stalling, Transmembrane transport

## Abstract

The phosphotransferase system glucose-specific transporter IICB^Glc^ serves as a central nutrient uptake system in bacteria. It transports glucose across the plasma membrane via the IIC^Glc^ domain and phosphorylates the substrate within the cell to produce the glycolytic intermediate, glucose-6-phosphate, through the IIB^Glc^ domain. Furthermore, IIC^Glc^ consists of a transport (TD) and a scaffold domain, with the latter being involved in dimer formation. Transport is mediated by an elevator-type mechanism within the IIC^Glc^ domain, where the substrate binds to the mobile TD. This domain undergoes a large-scale rigid-body movement relative to the static scaffold domain, translocating glucose across the membrane. Structures of elevator-type transporters are typically captured in either inward- or outward-facing conformations. Intermediate states remain elusive, awaiting structural determination and mechanistic interpretation. Here, we present a single-particle cryo-EM structure of purified, *n*-dodecyl-β-D-maltopyranoside-solubilized IICB^Glc^ from *Escherichia coli*. While the IIB^Glc^ protein domain is flexible remaining unresolved, the dimeric IIC^Glc^ transporter is found trapped in a hitherto unobserved intermediate conformational state. Specifically, the TD is located halfway between inward- and outward-facing states. Structural analysis revealed a specific *n*-dodecyl-β-D-maltopyranoside molecule bound to the glucose binding site. The sliding of the TD is potentially impeded halfway due to the bulky nature of the ligand and a shift of the thin gate, thereby stalling the transporter. In conclusion, this study presents a novel conformational state of IIC^Glc^, and provides new structural and mechanistic insights into a potential stalling mechanism, paving the way for the rational design of transport inhibitors targeting this critical bacterial metabolic process.

## Highlights

• Glucose transport in bacteria is facilitated by the IICB^Glc^ transporter.
• The cryo-EM structure of IIC^Glc^ in an intermediate state is reported.
• A ligand-induced wedging stalls the elevator-type transport cycle.
• This paves the way to the development of specific inhibitors for IICB^Glc^.

## Graphical abstract

**Figure:**
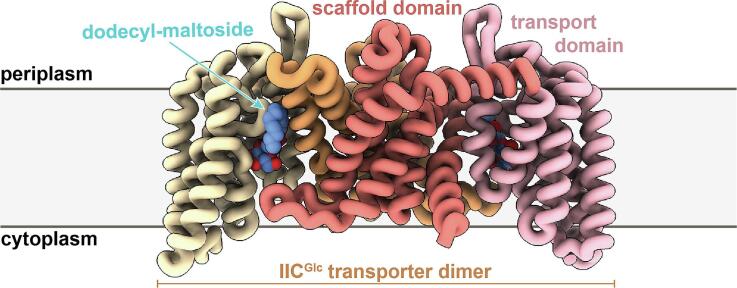

## 1 Introduction

**Fig. 1:**
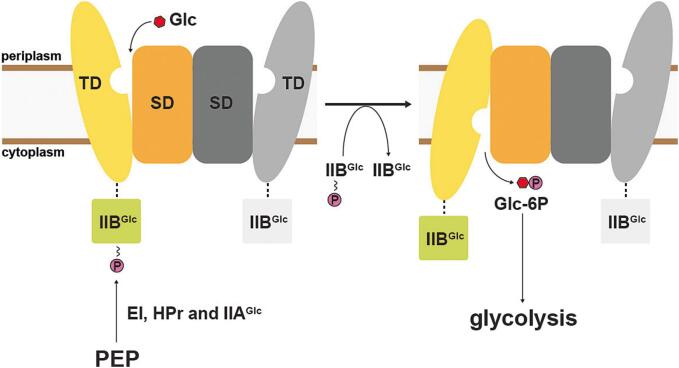
Scheme illustrating function and transport mechanism of IICB^Glc^. The membrane-embedded IIC^Glc^ protein domain comprises a scaffold (SD) and transport domain (TD), and mediates D-glucose (Glc; red hexagon) transport across the plasma membrane utilizing an elevator-type transport mechanism. Concomitant phosphorylation by the IIB^Glc^ protein domain, which is fused to IIC^Glc^ via a flexible linker (dashed line) converts the transported D-glucose to D-glucose-6-phosphate (Glc-6P), which directly enters the glycolytic metabolism. Energy is provided by phosphoenolpyruvate (PEP) and its phosphoryl group (letter P in a purple disc) is brought to IICB^Glc^ via the soluble enzyme cascade comprising the EI, HPr and IIA^Glc^ proteins.

Many bacteria possess the phosphoenolpyruvate-dependent phosphotransferase system (PTS), a distinct multi-component mechanism for the efficient uptake and utilization of carbohydrates as primary carbon source (Postma et al., 1993). Unlike other systems, the PTS operates via a group translocation mechanism, a coupled process in which the substrate is covalently modified concomitantly with its vectorial transport. The energy is derived from phosphoenolpyruvate, and its phosphoryl group is extracted and transferred through a series of soluble PTS components, namely the unspecific EI and HPr proteins, which are part of system I (Deutscher et al., 2006). System II, on the other hand, comprises substrate-specific proteins, including the integral membrane IIC proteins that act as the actual transmembrane transporters. IIC proteins are occasionally fused with other soluble phosphorylation cascade proteins, such as IIA and IIB, although these are also found in a detached form as independent soluble proteins in bacteria. IIC proteins are divided into four families, whereas the glucose/fructose/lactose superfamily is the largest (Nguyen et al., 2006, Saier et al., 2009, Saier et al., 2014). In addition to transporting and phosphorylating carbohydrates, components of the PTS play regulatory roles, integrating core carbohydrate metabolism with other essential cellular functions (Deutscher et al., 2014, Somavanshi et al., 2016). Given the preferential utilization of D-glucose (Glc; hereinafter referred to as glucose) by many bacteria, including *E. coli* (Carreon-Rodriguez et al., 2023), the glucose-specific PTS plays a central role and has long been used as the representative model to understand the principles and characteristics of the PTS. Especially the membrane-embedded components of the glucose-specific PTS have been the focus of extensive research (Gutknecht et al., 1998, Jeckelmann et al., 2011, Kalbermatter et al., 2015, Kalbermatter et al., 2017). We recently determined the structure of the glucose-specific IICB transporter (IICB^Glc^) from *E. coli*, which consists of the IIC^Glc^ permease fused to the IIB^Glc^ domain responsible for phosphoryl transfer (Roth et al., 2024). Interestingly, the IIB^Glc^ domain could not be resolved, suggesting an inherent flexibility of this domain. IIC^Glc^ operates via an elevator-type transport mechanism. As is typical for this mode of alternating access (Lee et al., 2017, Garaeva and Slotboom, 2020), the protein consists of a scaffold domain (SD), which promotes protein homodimerization, and a transport domain (TD), which contains the substrate-binding site (Fig. 1). During a transport cycle, the mobile substrate-bound transport domain moves as a rigid-body across the membrane, thereby transporting the substrate along. The recently published structures, which were determined from IICB^Glc^ transporters reconstituted in lipid nanodiscs, were captured in two physiologically relevant conformations: the inward- and outward-facing states (Roth et al., 2024). These structures provided first mechanistic insights into the transport mechanism. However, intermediate states between the inward- and outward-facing conformations remain to be elucidated.

Here, we determined the cryo-EM structure of IICB^Glc^ solubilized and purified in *n*-dodecyl-β-D-maltopyranoside (DDM). We present a high-resolution structure of the IIC^Glc^ transporter and identify a novel conformational state, in which the transport domain adopts an intermediate position between inward- and outward-facing conformation. In our search for the molecular basis of this intermediate state, well-resolved detergent molecules were observed, e.g., specifically binding to the glucose-binding site via the maltose head group. We propose a mechanism by which maltoside-based detergents stall the transporter. These findings provide valuable insights into the molecular basis of PTS-mediated carbohydrate transport in bacteria and pave the way for the development of innovative antibiotic compounds.

## 2 Results and discussion

### 2.1 Cryo-EM structure determination of DDM-solubilized IICB^Glc^

The glucose-specific PTS transporter IICB^Glc^ from *E. coli* was cloned and homologously overexpressed. Isolated bacterial membranes were solubilized, and the His-tagged transporter purified by nickel affinity chromatography using the detergent DDM in absence of a ligand. The isolated protein was pure and homogenous as documented by SDS-PAGE and size-exclusion chromatography, with an estimated molecular weight of ∼200 kDa (Suppl. Fig. 1). We then used cryogenic-electron microscopy (cryo-EM) to solve the structure. The vitrified sample showed well-distributed and easily discernible particles. A dataset comprising 9,036 movies was collected on a 300 kV Titan Krios microscope and single-particle analysis was performed (Suppl. Fig. 2a and b). Two-dimensional (2D) classification revealed averages of the particles in various orientations, and secondary structural features enclosed within a disordered detergent micelle became readily apparent (Suppl. Fig. 2c). An *ab-initio* reconstruction provided a preliminary Coulomb potential density map (hereinafter referred as density map), and further three-dimensional (3D) classification resulted in a set of particles, which was used for local refinement using a focussed soft mask surrounding the protein moiety. With imposed C_2_-symmetry, the final refinement yielded a 3D reconstruction at an overall resolution of 2.53 Å (Suppl. Fig. 2a and 3) according to the Fourier shell correlation (FSC) function using a threshold of 0.143 (gold-standard). The sharpened high-resolution map allowed us to build a structural model for residues 4–386 of the IICB^Glc^ protein. Additional densities could unambiguously be assigned to DDM molecules. Statistics on data acquisition, processing, model building and validation are provided in Table 1 and Suppl. Figs. 2 and 3.

**Table 1:** Statistics for cryo-EM data collection, model refinement and validation.

	**IIC^Glc^ intermediate state**
	PDB ID code: 9HNP EMD ID code: EMD-52311
**Data collection and processing**	
Microscope, camera	Krios G4, Falcon 4i
Magnification	165,000
Voltage (kV)	300
Electron exposure (e^–^/Å^2^)	35.04
Defocus range (μm)	−0.8 to −2.2
Pixel size (Å)	0.73277
Micrographs	9,036
Symmetry imposed	C_2_
Final particle images (no.)	563,331
Map resolution (Å)	2.53
FSC threshold	0.143

**Refinement**	
Model composition	
Non-hydrogen atoms	5924
Protein residues	766
Ligands	4
*B*-factors (Å^2^)	
Protein	45.10
Ligand	20.00
R.m.s.d.	
Bond lengths (Å)	0.015
Bond angles (°)	2.052
Validation	
MolProbity score	1.05
Clashscore	2.61
Poor rotamers (%)	0.34
Ramachandran plot	
Favored (%)	99.21 %
Allowed (%)	0.79 %
Disallowed (%)	0.00 %

Abbreviations used: e^–^, electron(s); EMD, electron microscopy data; FSC, Fourier shell correlation; kV, kilovolt; PDB, protein data bank; r.m.s.d., root mean square deviation.

### 2.2 Overall structure of IICB^Glc^

**Fig. 2:**
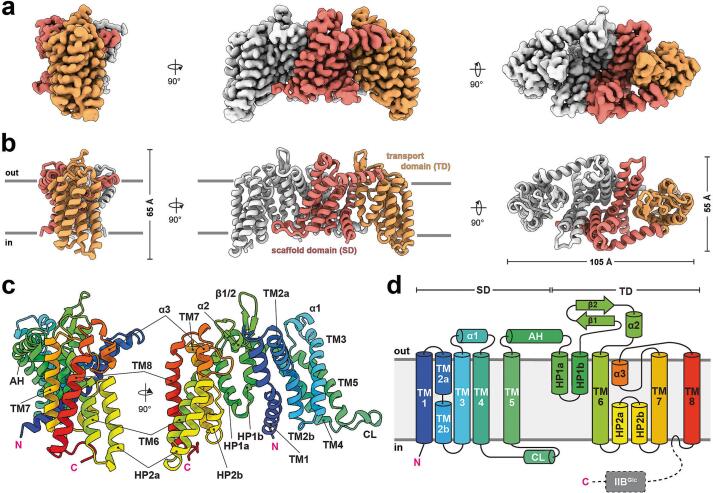
Overall structure and architecture of IIC^Glc^. (**a**) Three orthogonal views on the sharpened cryo-EM density map at 2.53 Å resolution, contoured at a threshold level of 0.04. One protomer is highlighted in colour and further divided into the scaffold domain (SD; red) and the transport domain (TD; orange). (**b**) The corresponding structural model, depicted as cartoon, is coloured to match the density map and is shown from the same perspectives as in (a). The boundaries of the plasma membrane are indicated. (**c**) Cartoon representation of the protomer, colour-coded using a rainbow scheme to highlight the major structural elements. Pink “N” and “C” denote amino- and carboxy-termini, respectively. (**d**) Topology plot for the protomer, consistently coloured as in (c). TM, transmembrane α-helix; HP, hairpin element; AH, amphipathic helix; CL, cytoplasmic loop.

IICB^Glc^ from *E. coli* is composed of the N-terminal IIC^Glc^ permease domain (approximatively 41.3 kDa) and the C-terminal catalytic IIB^Glc^ protein domain (approximatively 8.3 kDa), which are fused via a linker (approximatively 1.1 kDa). The high-resolution cryo-EM map reveals the detergent micelle-embedded dimeric transporter, consisting of two identical IIC^Glc^ protomers that form a homodimeric complex (Fig. 2a). The well-defined density abruptly vanishes beyond residue R386, which is putatively the start of the linker between IIC^Glc^ and IIB^Glc^. Notably, some side-view 2D class averages (i.e., views from the membrane plane) display diffuse density features extending from the more sharply curved, concave sides of the IIC^Glc^ transporter (Suppl. Fig. 2c), which correspond to the cytosolic side. The absence of pronounced cryo-EM density in both the 2D projections and the 3D reconstructions suggests a linker-mediated flexibility of the IIB^Glc^ domain, which may have hindered the determination of the complete structure of the IICB^Glc^ protein. This is in accordance with previous biochemical and structural findings (Buhr et al., 1994, Gutknecht et al., 1998, Roth et al., 2024).

The IIC^Glc^ transporter, with the majority of its mass embedded in the detergent micelle, exhibits an ellipsoid shape with dimensions of 105 × 55 × 65 Å (Fig. 2b). Its N- and C-termini are located on the intracellular side, and each protomer comprises eight transmembrane α-helices (TMs) and two hairpin (HP) elements. The protomers are further subdivided into a scaffold (SD) and a transport domain (TD), and consist of TMs 1–5 and TMs 6–8, respectively (Fig. 2). An amphipathic helix (AH) connects the two domains, while the cytoplasmic loop (CL) is in close spatial proximity to the opposite protomer. Other characteristic features include the short α-helices (α1-α3) and the prominent β-hairpin formed by two β-sheets (Fig. 2c and d). Both protomers interact tightly via a large hydrophobic interface area of about 1,990 Å^2^, formed by TMs 1–3 and TM5, and the CL (Suppl. Fig. 4a). Additional hydrogen bonds (N74/D75 and S54/G87) and a salt bridge (E50/K91) stabilize the homodimeric assembly (Suppl. Fig. 4b and c). In general, the topological architecture of IIC^Glc^ aligns with the previous descriptions of the nanodisc reconstituted structures (Roth et al., 2024). Similarly, the intrinsic flexibility of the linker from R386 onward remains consistent, regardless of the conformational state. This arginine residue was previously suggested to be an integral component of the conserved KTPGRED heptapeptide (Suppl. Fig. 5a, β-turn region) forming the proposed linker between the IIC^Glc^ and IIB^Glc^ protein domains (Lanz and Erni, 1998). However, in our structure, most of this peptide, specifically ^382^KTPGR^386^, adopts a well-folded β-turn conformation, serving as a structural element integral to the TD. Specifically, the β-turn motif lies on the cytoplasmic side of the TD and inserts within a cavity formed by TMs 6 and 8, and the HP2 (Suppl. Fig. 5b). The β-turn itself, which induces a reversal of the Cα chain, is formed by P384 and G385. The hydrogen bond between the backbone carbonyl group of T383 and the backbone amine of R386 further stabilizes this structure. The guanidium group of R386 is coordinated by backbone and a side chain interaction with D380, stabilizing this last resolved residue of IIC^Glc^ (Suppl. Fig. 5c). Collectively, these findings suggest that the flexible linker starts from R386 onward and the ^382^KTPGR^386^ motif is not part of the previously suggested linker.

### 2.3 IIC^Glc^ is in an intermediate conformational state

**Fig. 3:**
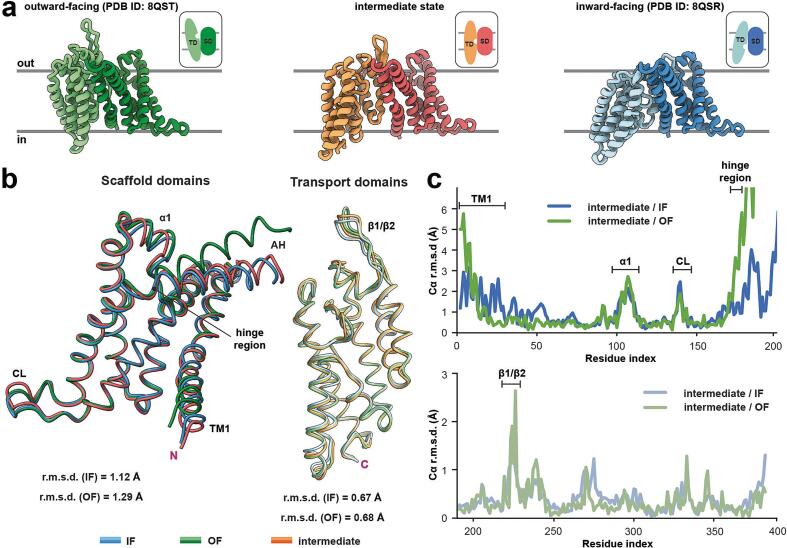
Characteristics of the IIC^Glc^ structure in the intermediate state conformation. (**a**) Side-to-side structural comparison of outward- (*left,* green; OF) and inward-facing (*right,* blue; IF) protomer models with the intermediate state (*middle,* orange) from this study. Scaffold domains (SDs) and transport domains (TDs) are colored in dark and bright shades, respectively. The insets display schematic representations of the elevator-type conformational changes, with the ligand binding pocket highlighted by a cavity in the TD. The membrane plane is also indicated. (**b**) Structural alignment of SDs (residues 4–172) and TDs (residues 191–386) in IF, OF and intermediate states with indicated root mean square deviations (r.m.s.d.). The amphiphilic helix (AH) connecting SD with the TD, and the termini (pink “N” and “C” for amino- and carboxy-termini, respectively) are indicated. (**c**) Pairwise Cα deviation plots of individually aligned SDs (*top*) and TDs (*bottom*) of IF/OF protomers with the intermediate state for the identification of structural differences.

We previously proposed an elevator-type transport mechanism for IIC^Glc^, in which the TD undergoes a rigid-body movement relative to the SD. As a result, the substrate-binding site within the TD translocates along the membrane normal and undergoes a slight rotation, facilitating alternating access to either the cytoplasm or the periplasm. Visual examination of the current structure with the previously published IIC^Glc^ structures in the inward- (IF) and outward-facing (OF) states reveals considerable differences in the position of the TDs (Fig. 3a). This finding suggests that the current structure may represent an intermediate conformation. To further analyze the conformation of this elevator-type transporter, the protomers were dissected into their rigid-body components, namely the SD and TD, for structural comparison. The isolated SD shows good alignment with both the IF and OF model SDs, with average root mean square deviation (r.m.s.d.) values of 1.12 Å and 1.29 Å, respectively. Similarly, the isolated TD aligns closely with the IF and OF model TDs, exhibiting average r.m.s.d. values of 0.67 Å and 0.68 Å, respectively (Fig. 3b). Differences in the SDs are particularly evident in TM1, α1 and the CL, whereas in the TD, only the β-hairpin exhibits a notably distinct arrangement (Fig. 3c). When the published models (Roth et al., 2024) are globally aligned to the SD of the current structure, a notable structural shift in the vertical position, i.e., along the membrane normal, of the TD becomes readily apparent. In the OF state, the TD extends into the periplasmic space, while in the IF state, it tilts toward the cytosol. In contrast, the TD of the current structure is positioned within the membrane plane, representing an intermediate state between IF and OF (Fig. 3a). The CL, which interacts with the TD of the opposite protomer in the IF and OF structures, appears highly flexible, as indicated by its high *B*-factor values (Suppl. Fig. 3g). A comparison of the hinge point around the double proline motif (P173/P174), which connects the SD and TD, shows higher similarity to the IF state up to the beginning of the TD around HP1a. Also, TM1 is closer to the IF conformation (Fig. 3b). Collectively, the structural comparison with the IF and OF structures reveals that the TD in the current structure is located halfway between inward- and outward-facing states, representing an intermediate state in the transport cycle.

### 2.4 Molecular basis for the interaction of IIC^Glc^ with the maltoside-based detergent DDM

**Fig. 4:**
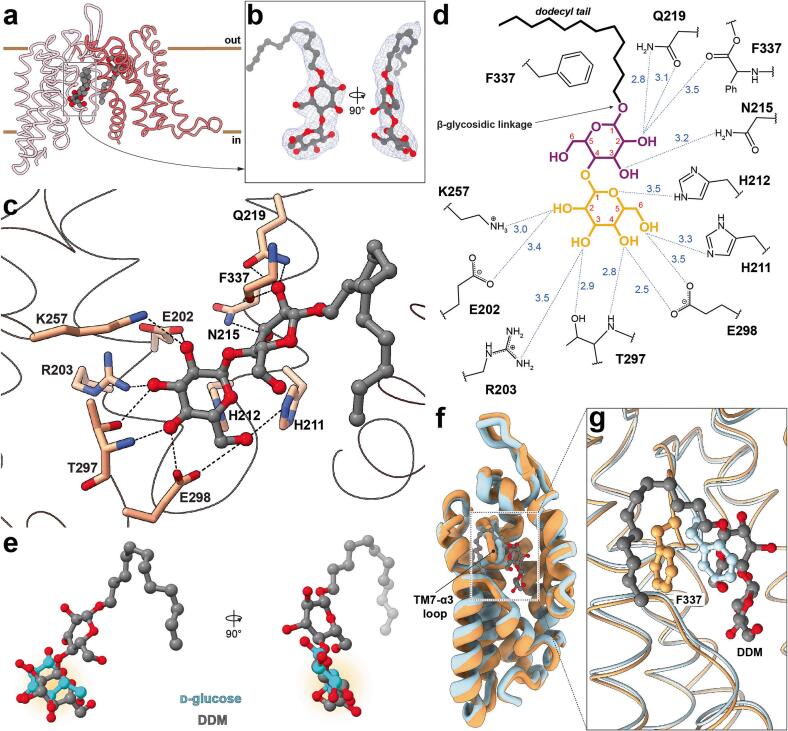
Interaction of *n*-dodecyl-β-D-maltopyranoside (DDM) with the substrate-binding site of IIC^Glc^ and the induced shift of the thin gate F337. (**a**) Model of the monomer (as viewed from the membrane plane) with location of the resolved DDM molecules. (**b**) Orthogonal view on the cryo-EM density (contour level of 0.05) and the fitted model of the boxed DDM molecule in (a), which is specifically coordinated by the IIC^Glc^ substrate-binding site. (**c**) Molecular basis for DDM binding within the substrate-binding site and specific hydrogen bond interactions (≤3.5 Å, represented as dashed lines). (**d**) Two-dimensional interaction diagram with indicated hydrogen bond distances (dashed lines, in Å). The foremost glucopyranosyl moiety (orange) of the DDM molecule's maltoside head group is specifically coordinated by residues in the substrate-binding site. The second glucopyranosyl moiety (purple) and the dodecyl tail (black) extend out of the cavity at the scaffold/transport domain interface. Note that the alkyl tail hydrophobically interacts with a variety of amino acid residues, and only the most prominent hydrophobic interaction with the phenyl group of F337 is shown for the sake of clarity. Ph, phenyl group of F337. (**e**) Superposition of the transport domains in the glucose-bound inward-facing state (PDB ID: 8QSR) with the DDM-bound intermediate reveals a comparable recognition mode for glucose (light blue) and the glucopyranoside moiety (highlighted with an orange shadow) of DDM (grey). (**f**) Model of the transport domains in DDM- (orange) and glucose-bound state (light blue, PDB ID: 8QSR). The location of the substrate-binding site occupied by DDM (grey) is boxed. (**g**) A zoom-in view reveals the shifted TM7-α3 loop, harbouring the thin gate residue F337. The DDM's alkyl tail wraps around the phenyl group of F337.

In investigating the cause of this intermediate arrangement of the TD, a closer examination of the cryo-EM density map revealed two tightly interacting DDM molecules within IIC^Glc^ protomers (Suppl. Fig. 3f). One of them occupies a surface-exposed cavity formed by the AH at the SD/TD interface on the periplasmic side of the transporter. Here, the maltoside head group is stabilized by the side chain of Q221 and the backbone carbonyl of G53, whereas the alkyl tail is involved in multiple hydrophobic interactions (Suppl. Fig. 6). Interestingly, the other DDM molecule was found trapped in the IIC^Glc^ substrate-binding site located in the TD (Fig. 4a and b). Its maltose head group, which is a glucose disaccharide, is specifically bound via the foremost glucopyranosyl moiety, i.e., the glucose unit more distant relative to the alkyl tail. Residues within the substrate-binding site form numerous hydrogen bonds to the hydroxyl groups (OH) and the ring oxygen (O_R_) of this glucopyranosyl moiety (Fig. 4c). Specifically, E298 interacts with OH-4 and OH-6 (OH-6 is additionally coordinated by H211), T297 with OH-3 and OH-4, R203 with OH-3 and both K257 and E202 engage with hydrogen bonds with OH-2, while the O_R_ is in contact with H212. The second glucopyranosyl moiety is α(1 → 4)-linked to the first one and is in a slightly twisted configuration. Q219 and the backbone carbonyl of F337 coordinate its OH-2 and N215 coordinates the OH-3 groups (Fig. 4d). At position 1 of the second glucopyranosyl moiety, the dodecyl alkyl tail is attached via a β-glycosidic linkage and extends from the substrate-binding site. A variety of hydrophobic interactions stabilize the alkyl tail, primarily mediated by I28, G31, V32 and F337.

The specific binding of maltose and the glycosidic detergent octyl-glucoside by IIC^Glc^ was previously shown (Jeckelmann et al., 2011), demonstrating substantial competitive effect of these ligands on the binding of [^3^H]glucose in scintillation proximity assay experiments. In line with our previous results, modifications at OH-1 are tolerated as demonstrated by 1-methyl-glucose, 1-deoxy-glucose and, most notably, the glucose disaccharide maltose (Roth et al., 2024). A comparison of the binding mode of the foremost glucopyranosyl moiety in DDM with the glucose substrate in the occluded IF state reveals a comparable, yet slightly rotated arrangement. This subtle difference may be attributed to the steric constraints imposed by the remaining portion of the DDM molecule (Fig. 4e). In a broader context, the loop between TM7 and α3 is shifted by several angstroms in the DDM bound state, and especially the prominent phenyl group of F337 appears considerably displaced (Fig. 4f and g).

### 2.5 Potential stalling mechanism

**Fig. 5:**
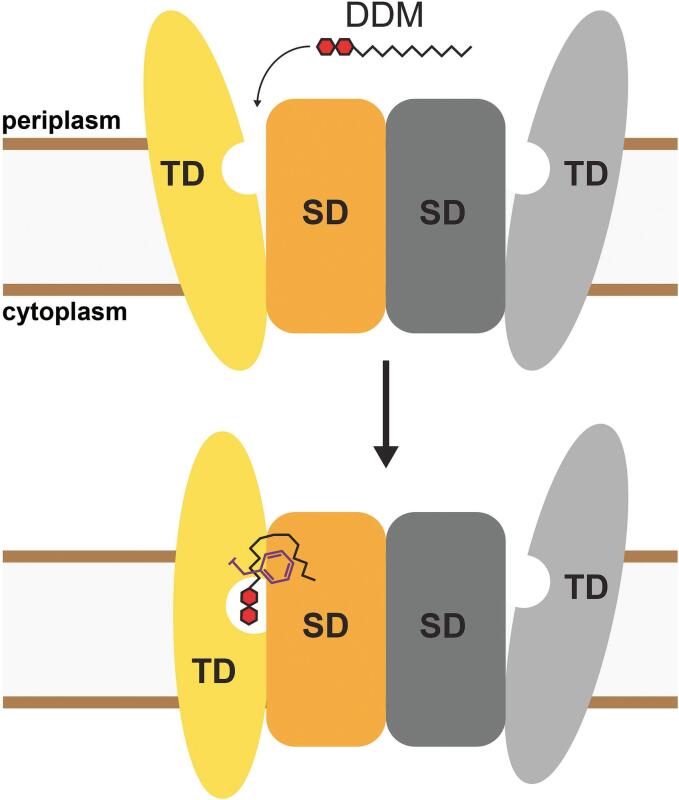
Proposed mechanism of DDM-induced IIC^Glc^ transporter stalling. A DDM molecule specifically binds to the substrate-binding site of the outward open conformation of the IIC^Glc^ transporter via its maltoside head group. This interaction triggers a conformational change toward the inward-facing state. The elevator-type movement involves both a physical translocation of the transport domain (TD) along the membrane normal and a rotation of the binding pocket relative to the static scaffold domain (SD). However, the bulky alkyl chain of the DDM molecule laterally displaces the thin gate residue F337 (purple) and the associated TM7-α3 loop, effectively acting as a wedge. This displacement stabilizes the transporter in a stalled intermediate state conformation.

The observed intermediate state of the IIC^Glc^ TD, combined with the discovery of a lodged DDM molecule, suggests a stalled state within the transport pathway. From one perspective, the specific recognition of the maltose head group of DDM within the substrate-binding site, coupled with the bulky nature of its alkyl tail, appears to play a key role in stabilizing this state. On the other hand, the TM7-α3 loop harbouring residue F337 was observed in an unusual horizontally shifted position. Interestingly, we previously identified F337 as a thin gate, shielding the polar substrate-binding site. Occlusion is expected upon substrate binding and is a prerequisite for the formation of the outward-facing, occluded state, which then leads to the large-scale elevator-type conformational rearrangement to inward-facing states. Indeed, while the maltose head group competitively occupies the substrate-binding site, the bulky alkyl tail of DDM partially wraps around the phenyl group of F337, interacts hydrophobically with residues from the SD and displaces the entire TM7-α3 loop, thereby preventing the full occlusion of the thin gate. Therefore, DDM figuratively acts as a wedge, supported by the thin gate F337. These findings suggest that the elevator-type sliding motion across the SD/TD interface is impaired, resulting in a blockage of the transport cycle and gives rise to this DDM-induced intermediate stalled state (Fig. 5).

## 3 Conclusion

The PTS is the major glucose transport system in bacteria and IICB^Glc^ acts as the membrane transporter. Here, we determined its structure using cryo-EM, revealing an intermediate conformation between inward- and outward-facing state in the elevator-type transport cycle, providing valuable mechanistical insights. A DDM detergent molecule was found to be trapped within the substrate-binding site with its specifically-bound glucopyranoside moiety. Due to the bulky nature of this ligand, it acts as a kind of wedge and induces a shift of the thin gate residue F337, thereby stalling the transporter in this intermediate state. These results provide a molecular basis for the development of IICB^Glc^ targeting molecules, which in turn might lead to the development of novel bactericidal or bacteriostatic agents to fight the arising antibiotic resistances. Indeed, a PTS inhibitor has recently been shown to exert an antibacterial effect by disrupting energy metabolism pathways (Tan et al., 2020) and it remains to be demonstrated if molecules specifically targeting IICB^Glc^ have a similar effect.

## 4 Materials and methods

### 4.1 Construct, expression and membrane isolation

The IICB^Glc^ transporter from *E. coli* (UniProt: P69786) was cloned into the pZUDF plasmid (Ilgü et al., 2014), encoding the full-length protein with a C-terminal 3C protease cleavage site followed by a deca-histidine tag. The protein was expressed as described previously (Roth et al., 2024). Briefly, *E. coli* strain BW25113(DE3) ΔarcBΔptsG (made in-house based on (Baba et al., 2006)) was transformed with the pZUDF-IICB^Glc^ construct and was cultivated in LB (Luria Bertani) medium supplemented with 100 μg/mL ampicillin. Cells were grown in an incubator shaker (Multitron, Infors HT) at 250 rpm and 37 °C, and overexpression was induced by addition of 200 µM isopropyl-β-D-1-thiogalactopyranoside (IPTG; final concentration) for 4 h. Cells were harvested by centrifugation (10,000 × g, 10  min, 4 °C) and washed once in lysis buffer (50 mM Tris-HCl pH 8.0, 300 mM NaCl). Resuspended cells were lysed by five passages through a Microfluidizer MP-110 (Microfluidics) at 1,500  bar and cell debris were removed by centrifugation (10,000 × g, 10  min, 4 °C). The membranes residing in the supernatant were isolated and washed once in membrane buffer (20 mM HEPES-NaOH [4-(2-hydroxyethyl)-1-piperazineethanesulfonic acid] pH 8.0, 300 mM NaCl) by ultracentrifugation (100,000 × g, 30 min, 4 °C). The final membrane pellet was resuspended in membrane buffer using a Potter-Elvehjem homogenizer, adjusted to a concentration of 200 mg/mL (w/v), aliquoted, flash-frozen in liquid nitrogen and stored at −80 °C until further use.

### 4.2 Purification of IICB^Glc^

For purification, 400 mg of membranes were solubilized in 7 mL membrane buffer supplemented with 2 % (w/v) *n*-dodecyl-β-D-maltopyranoside (DDM, Glycon) and 5 mM β-mercaptoethanol (β-ME) for 90 min at 8 °C. Unsolubilized material was removed by ultracentrifugation (100,000 × g, 30  min, 4 °C) and the supernatant was diluted 1:1 with wash buffer (20 mM HEPES-NaOH pH 8.0, 300 mM NaCl, 5 mM β-ME, 20  mM imidazole and 0.03 % (w/v) DDM) to reduce the detergent concentration. The protein was then incubated with 500 µL equilibrated Ni^2+^-NTA resin (Qiagen) for 90 min at 8 °C. The slurry was subsequently transferred into a disposable column (Promega) and washed sequentially with 40 column volumes of wash buffer and five column volumes of size-exclusion chromatography (SEC) buffer (20 mM HEPES-NaOH pH 8.0, 5 mM β-ME, 150 mM NaCl and 0.03 % (w/v) DDM). The lower part of the column containing the resin was cut using a razor blade and sealed with parafilm. The IICB^Glc^ protein was specifically eluted by on-column cleavage (Hirschi and Fotiadis, 2020) using home-made His-tagged human rhinovirus (HRV) 3C protease (final concentration of 0.4 mg/mL). Cleavage was performed in 450 µL SEC buffer for 90 min at 8 °C and the eluted protein was then concentrated using a 0.5 mL centrifugal concentrator with a 100 kDa molecular weight cut-off (Amicon). Further purification was performed by SEC using a Superdex 200 10/300 column mounted on an Äkta pure system (Cytiva). Peak fractions corresponding to a calibrated molecular weight (high molecular weight calibration kit, Cytiva) of ∼200 kDa were combined, concentrated about two-fold using a centrifugal concentrator (same as above) and centrifuged (20,000 × g, 10  min, 4 °C) to remove possible aggregates. The final protein concentration was determined spectrophotometrically using a NanoDrop OneC (Thermo Scientific) and a molar extinction coefficient of 53,860 M^−1^·cm^−1^ at 280 nm. The purity was assessed by SDS-PAGE on 13.5 % SDS/polyacrylamide gels stained with Coomassie Brilliant Blue R-250 dye. The Precision Plus Protein™ Dual Color Standard (BioRad) was utilized as molecular weight marker.

### 4.3 Cryo-EM sample preparation and data acquisition

Cryo-EM samples were prepared using glow-discharged (PELCO easiGlowTM system) Quantifoil copper R2/1 200-mesh holey carbon grids and a Mark IV Vitrobot (FEI) set to 100 % humidity at 4 °C. After applying 3 µL of the purified protein at 6.2 mg/mL, grids were blotted for 4 s using a blot force of −4 and vitrified by plunging into liquid ethane cooled by liquid nitrogen. Grids were screened on a FEI Tecnai F20 field emission gun electron microscope operated at 200 kV and equipped with a Falcon III camera. Automated high-resolution data acquisition was performed on a Titan Krios G4 equipped with E-CFEG (energy-filtered cold field emission gun) operated at 300 kV acceleration voltage, a post-column Selectris X energy filter (set to 10 eV) and a Falcon 4i direct electron detector (Thermo Fisher) operated in counting mode. Aberration-free image shift (AFIS) and fringe-free imaging (FFI) allowed circular collection of nine micrographs per foil hole assisted by the EPU software, which yielded a throughput of ∼650 images per hour. Micrographs were acquired in the electron-event representation (EER) format at a nominal magnification of ×165,000 (corresponding to a pixel size of 0.73277 Å), with a constant exposure duration of 3.0 s (resulting in an adjusted dose of ∼35.04 e^-^/Å^2^, split in 860 fractions) in a target defocus range of −0.8 to −2.2 µm.

### 4.4 Cryo-EM data processing

Single-particle analysis data processing was performed using the software package cryoSPARC v.4.1.0 (Punjani et al., 2017). In total 9,036 movies were imported and subjected to patch CTF (contrast transfer function) estimation and patch motion correction (9 × 9 patches), resulting in movie stacks of 40 fractions. Micrographs with poor statistics were removed (i.e., CTF fit > 5 Å, according to average intensity, total full-frame motion distance > 20 pixels, astigmatism > 280 Å) resulting in a set of 7,881 micrographs. References for template picking were generated from ∼500 manually picked particles, and ultimately 6,831,987 particles were automatically picked and extracted (extraction box size of 384 pixels binned to 96 pixels) from the full dataset. Consecutive rounds of 2D classification were used to remove bad picks and the remaining 1,258,959 particles in class averages showing high-resolution features were used for multi-reference *ab-initio* 3D reconstruction. The visually best class with recognizable secondary structural features was selected as a reference map and corresponding particles were subjected to two rounds of heterogeneous refinement. The remaining 563,331 particles were aligned using a homogenous refinement and re-extracted (extraction box size of 360 pixels binned to 260 pixels), followed by alignment using a non-uniform refinement (Punjani et al., 2020). A soft mask enclosing the protein density and excluding the detergent micelle was generated in ChimeraX (Pettersen et al., 2021) using the *molmap* command and used for local refinement (with per-particle scale minimization and Gaussian prior enabled). Global (refinement of higher-order aberrations) and local CTF (per-particle defocus optimization) corrections were performed, and a local refinement with C_2_-symmetry imposed yielding the final 3D reconstruction with a global resolution of 2.53 Å according to FSC (Fourier shell correlation) at the 0.143 gold-standard threshold criterion. Local resolution and directional FSC were computed using the dedicated job types within cryoSPARC. The density half-maps were sharpened using the *highres* learning model within the DeepEMhancer (Sanchez-Garcia et al., 2021) environment for protein model building and density visualization.

### 4.5 Model building and validation

As an initial template, the monomer of the previously published model for the inward-facing IIC^Glc^ (PDB ID: 8QSR) was used. The scaffold and transport domains of the monomer were separately fit into the sharpened cryo-EM density map using ChimeraX and provisionally adjusted using the interactive molecular-dynamics flexible fitting of the Isolde plugin (Croll, 2018). In an iterative process, the model was manually adjusted in Coot v0.9.6 (Emsley et al., 2010) and refined using the *phenix.real_space_refine* implemented in Phenix v1.19.2 (Adams et al., 2010, Afonine et al., 2018) until the statistics were satisfactory. Models and restraints for β-DDM molecules were generated using eLBOW (Moriarty et al., 2009) and they were manually built in the yet unmodelled densities with aid of the unsharpened density. The refined model was then used to build the C_2_-symmetric homodimer and finalized by a last round of refinement in Phenix. Statistics were validated using the cryo-EM validation tool in Phenix and the MolProbity server (Davis et al., 2007) (https://molprobity.biochem.duke.edu/).

### 4.6 Miscellaneous

Cryo-EM density maps and atomic models were visualized in ChimeraX, and figures were prepared using Adobe Illustrator (Adobe Inc.). The PDBePISA server (Krissinel and Henrick, 2007) (https://www.ebi.ac.uk/pdbe/pisa/) was used for interface analysis. Structural alignments, r.m.s.d. calculations and pairwise deviations were performed in ChimeraX (Pettersen et al., 2021). The OPM server (Lomize et al., 2022) (https://opm.phar.umich.edu/ppm_server3_cgopm) was used for membrane placement. Conservation analysis was performed on the ConSurf server (Ashkenazy et al., 2016) (https://consurf.tau.ac.il/) using default parameters.

### CRediT authorship contribution statement

**Patrick Roth:** Writing – review & editing, Writing – original draft, Methodology, Investigation, Formal analysis, Data curation. **Dimitrios Fotiadis:** Writing – review & editing, Writing – original draft, Resources, Project administration, Funding acquisition, Conceptualization.

## Declaration of competing interest

The authors declare the following financial interests/personal relationships which may be considered as potential competing interests: Dimitrios Fotiadis reports financial support was provided by Swiss National Science Foundation. Dimitrios Fotiadis reports financial support was provided by UniBern Forschungsstiftung. If there are other authors, they declare that they have no known competing financial interests or personal relationships that could have appeared to influence the work reported in this paper.

## Data Availability

The cryo-EM density map used for model building (along with the unsharpened map, half maps and the mask used for refinement), and the associated model have been deposited in the Electron Microscopy Data Bank (EMDB) and Protein Data Bank (PDB), respectively, with the accession IDs as follows: EMD-52311 and PDB 9HNP.
