# Supplementary material for: Cryo-EM structure of a phosphotransferase system glucose transporter stalled in an intermediate conformation
Source: J Struct Biol X. 2025 Mar 5;11:100124. doi: 10.1016/j.yjsbx.2025.100124 (PMC11930441; doi:10.1016/j.yjsbx.2025.100124)
Supplement: Supplementary Data 1 [file mmc1.pdf]

Supplementary Information

Cryo-EM structure of a phosphotransferase system glucose transporter stalled in an intermediate conformation

Patrick Roth, Dimitrios Fotiadis*

Institute of Biochemistry and Molecular Medicine, Medical Faculty, University of Bern, Bern, Switzerland

* Corresponding author: dimitrios.fotiadis@unibe.ch

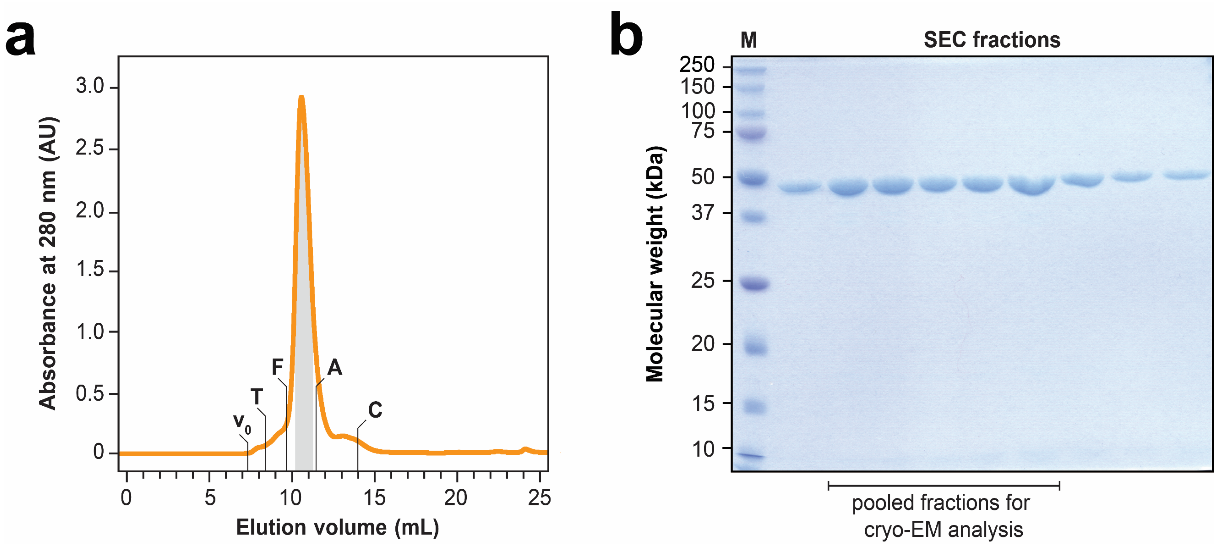

Suppl. Figure 1: Biochemical characterization of IICB^{Glc} solubilized and purified in *n*-dodecyl-β-D-maltopyranoside (DDM). (**a**) Preparative size-exclusion chromatography (SEC) elution profile of IICB^{Glc} using a Superdex 200 10/300 column. The pooled peak fractions used for cryo-EM sample preparation are indicated in grey. Elution volumes of the void (v_0) and different molecular weight (M_w) markers (T, thyroglobulin [M_w ~669 kDa]; F, ferritin [M_w ~440 kDa]; A, aldolase [M_w ~158 kDa] and C, conalbumin [M_w ~75 kDa]) are indicated; the elution peak corresponds to an estimated M_w of ~200 kDa, consistent with the expected mass for the ternary complex consisting of dimeric IICB^{Glc}, lipids and the DDM micelle. (**b**) Protein of the collected elution peak fractions from SEC were individually resolved on a 13.5% SDS-PAGE gel and visualized by staining with Coomassie Brilliant Blue R-250. Fractions are ordered from left to right by increasing elution volume from SEC. "M" denotes the molecular weight marker lane. Purified IICB^{Glc} protein migrates near the 50 kDa marker. The collected fractions from the SEC peak in (a) that were used for cryo-EM analysis are indicated.

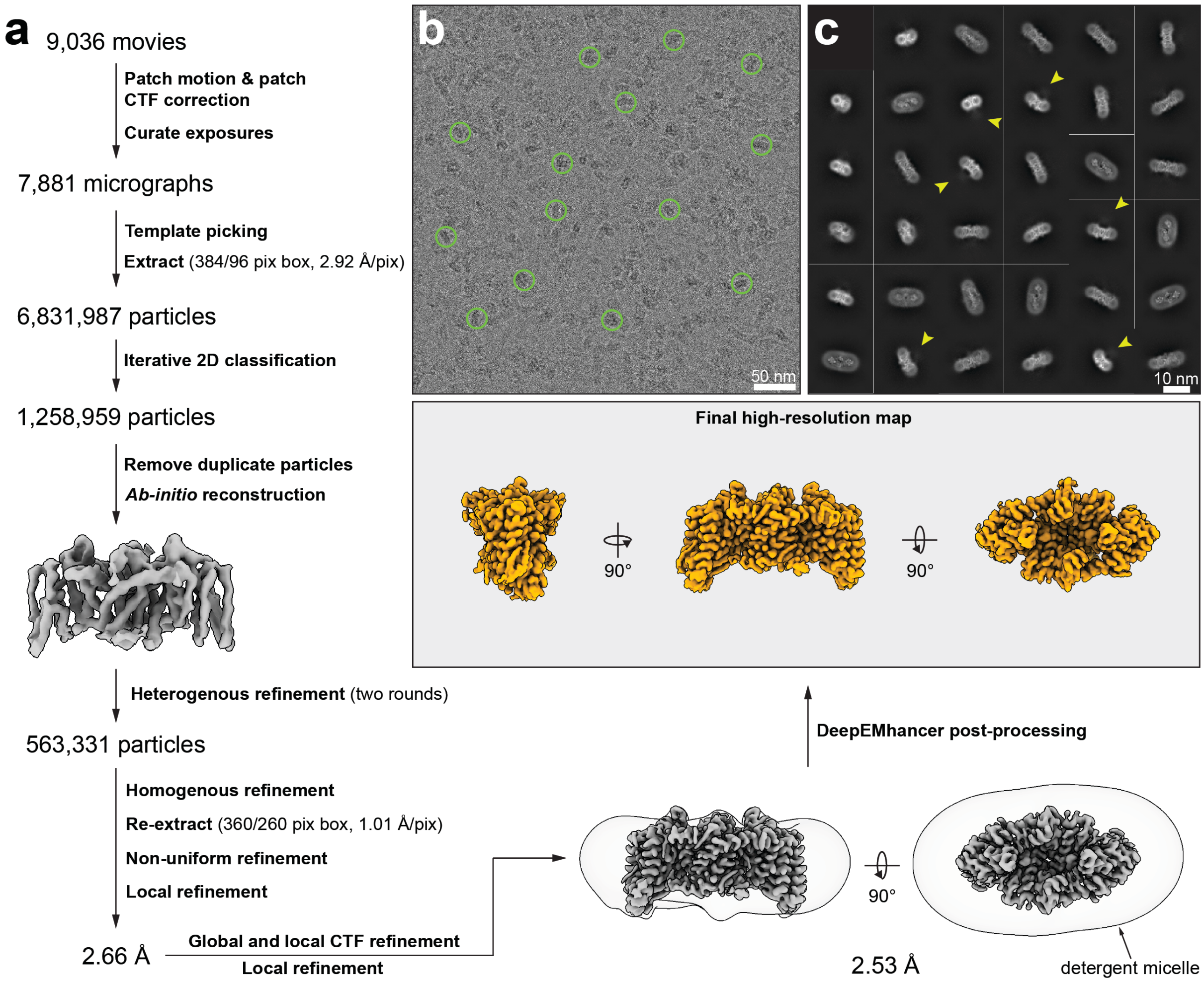

Suppl. Figure 2: Cryo-EM single-particle analysis and 3D reconstruction. (**a**) Data processing workflow leading to the 3D reconstruction of IICB^{Glc} at 2.53 Å resolution (according to the gold-standard 0.143 threshold criterion). Further details on data processing are provided in the materials and methods section. The final high-resolution map (colored in orange) is shown from three different views. Deposition identifiers for the protein data bank (PDB) and electron microscopy database (EMD) are 9HNP and EMD-52311, respectively. (**b**) Representative electron micrograph (at 1.8 µm defocus) recorded on a Titan Krios G4 microscope equipped with an energy filter (set to 10 eV) and a Falcon 4i camera. Clearly visible particles are highlighted with green circles. (**c**) Selected two-dimensional (2D) class averages after iterative 2D classification. Diffuse densities on the concave faces of characteristic side-view classes of IIC^{Glc} dimers, potentially corresponding to IIB^{Glc}, are marked with yellow arrowheads.

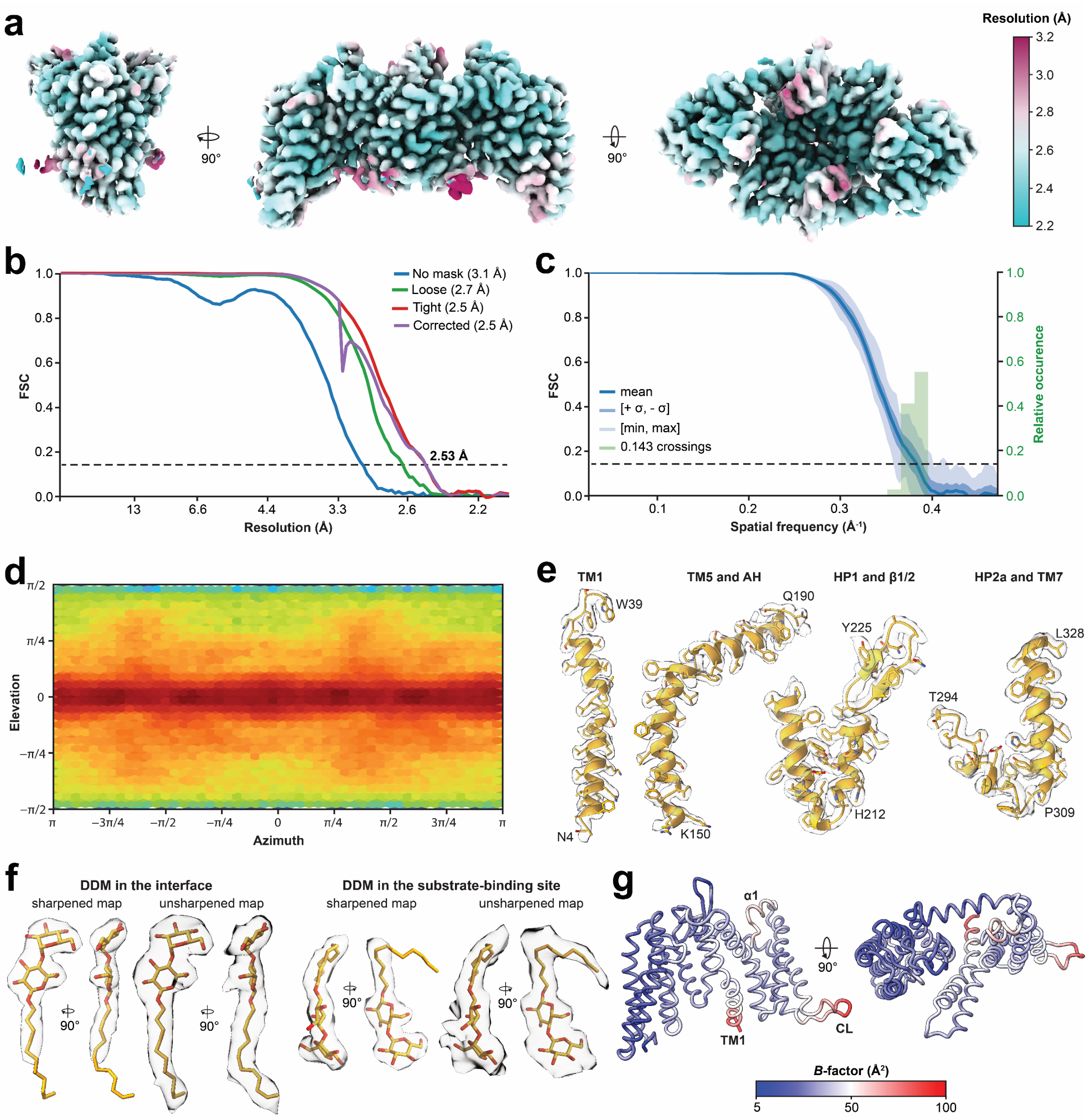

Suppl. Figure 3: Cryo-EM map density analysis and validation. (**a**) Orthogonal views on the final high-resolution cryo-EM map coloured by the computed local resolutions. (**b**) Fourier-shell correlation (FSC) plot of the final local refinement with imposed C_2 symmetry and the corresponding (**c**) conical FSC (cFSC) plot indicating the directional signal content. (**d**) The viewing direction distribution plot illustrates the number of particles at specific elevation and azimuth angles used in the final reconstruction. (**e**) Representative model-to-map fit for selected structural elements, e.g., transmembrane α-helices (TMs), hairpins (HPs), amphipathic helix (AH) and β-sheet (β1/2). The model is depicted as a ribbon representation (light orange) and the cryo-EM density map as a transparent surface (contour level of 0.112). For reference, selected amino acid residues are labelled according to their sequence positions. (**f**) Model-to-map fit of the two bound DDM molecules in the sharpened and unsharpened maps (contour level of 0.05). (**g**) Cartoon model of the protomer coloured by computed *B*-factor.

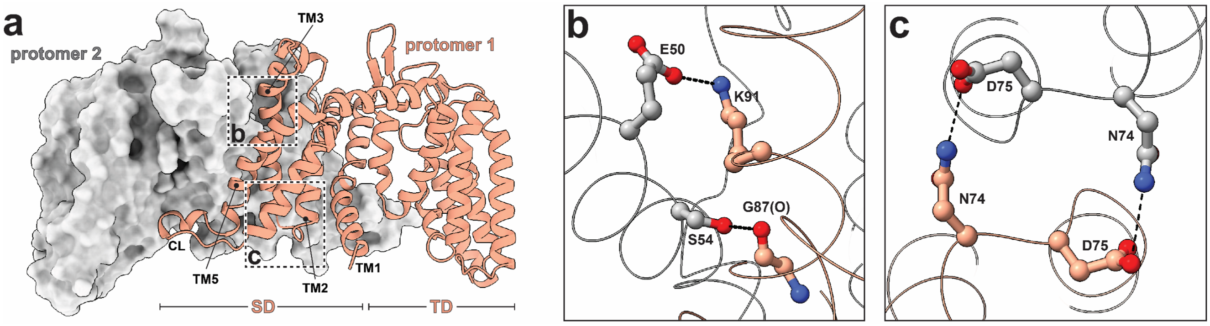

Suppl. Figure 4: Protomer arrangement and polar dimer interface contacts in the IIC^{Glc} intermediate state structure. (**a**) View on the dimeric assembly of IIC^{Glc} in the intermediate conformational state. One protomer is depicted as cartoon model (pale pink, *protomer 1*) and the other protomer is rendered as a grey surface (*protomer 2*). Location of scaffold (SD) and transport domains (TD) of *protomer 1* are indicated for guidance, and structural elements involved in oligomerization are labelled, i.e., TMs1-3 and TM5, and the cytoplasmic loop (CL). Panels (**b**) and (**c**) present close-up views of the regions marked by dashed boxes in panel (a), highlighting polar interactions (distances ≤3.5 Å) between protomers: E50 and K91 form a salt bridge, and the other shown interactions are hydrogen bonds. The "O" in brackets in panel (b) denotes the backbone carbonyl of glycine 87.

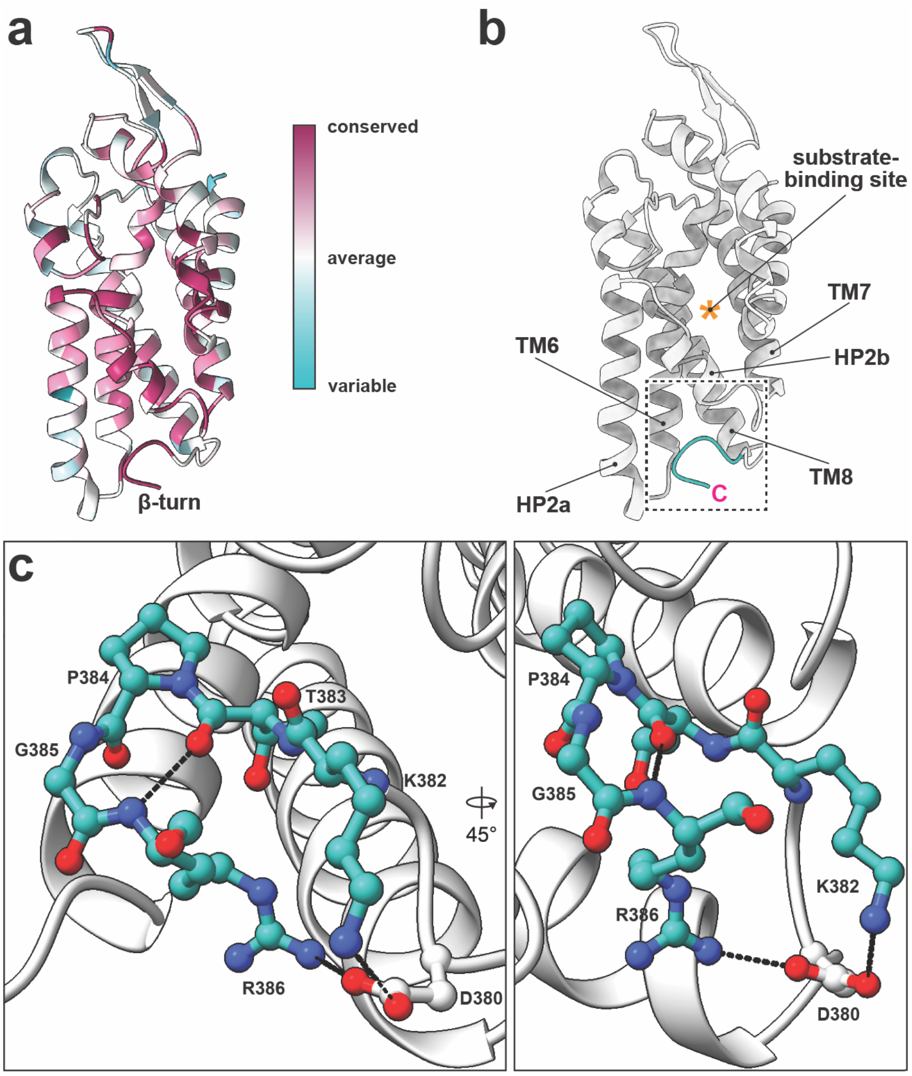

Suppl. Figure 5: Conservation, structure and location of the resolved part of the conserved KTPGRED motif in $IICB^{Glc}$. (**a**) Conservation analysis (https://consurf.tau.ac.il/) of the transport domain reveals a high evolutionary conservation of the β-turn motif. (**b**) Location of the resolved residues (cyan) of the C-terminal KTPGRED motif (residues 382–388) within the TD. The localization of the substrate-binding site is marked with a yellow asterisk. (**c**) Structure and polar interactions (distances ≤3.5 Å) of the resolved ^{382}KTPGR^{386} moiety forming an ordered β-turn structure. The flexible linker connecting IIC^{Glc} and IIB^{Glc} protein domains appears to start from R386 onwards: No density was resolved in our IIC^{Glc} structure for the subsequent protein amino acids, indicating their flexibility.

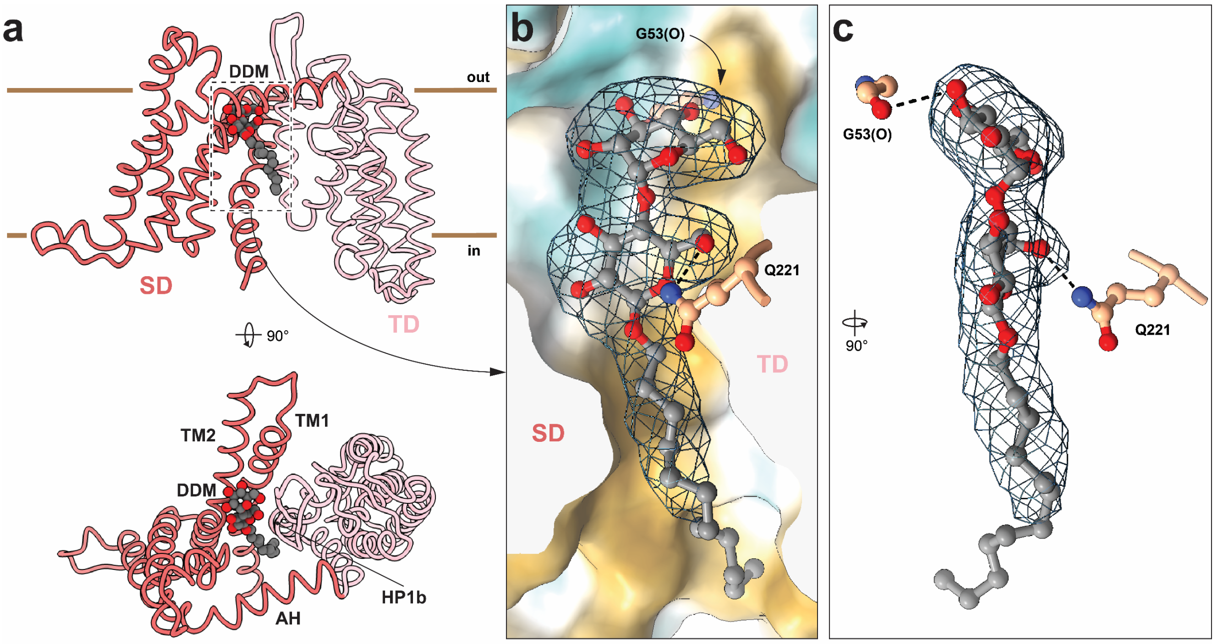

Suppl. Figure 6: Location and interactions of the DDM molecule identified at the SD-TD interface. (**a**) The cavity where the DDM molecule is bound, is formed by the amphipathic helix (AH), the scaffold domain (SD, red) and the transport domain (TD, pink). For clarity, only one protomer is shown and the membrane boundaries are indicated. The structure is viewed from the membrane plane (top portion of the panel). Secondary structure elements in the vicinity are labeled in the view seen from the periplasm (bottom portion of the panel). (**b**) Close-up view of the cavity (indicated by the dashed box in panel (a)) containing the DDM molecule. The protein surface is coloured based on hydrophobicity, with cyan representing hydrophilic regions and goldenrod indicating lipophilic patches. Residues involved in polar interactions with the maltoside head group of the DDM molecule (grey) are highlighted. (**c**) Orthogonal view (relative to panel (b)) of the bound DDM molecule with indicated hydrogen bonds between specific maltoside hydroxyl groups and the carbonyl oxygen of glycine 35 (G53(O)) as well as the side chain of Q221. The cryo-EM density for DDM in (b) and (c) is shown as mesh and is rendered at a contour level of 0.05.
